# Memoir upon the Peripheral Terminations of Motor Nerves in the Animal Series

**Published:** 1869-02-15

**Authors:** S. Trinchese

**Affiliations:** From La Gazette Médicale


### CHICAGO MEDICAL JOURNAL.

VOL. XXVI.—FEBRUARY 15, 1869.—No. 4.

### TRANSLATIONS.

TRANSLATED EXPRESSLY FOR THIS JOURNAL, BY W. HAY, M.D., ASSOCIATE EDITOR.

### *Memoir upon the Peripheral Terminations of Motor Nerves in the Animal Series.*

BY S. TRINCHESE—FROM LA GAZETTE MÉDICALE.

*Conclusions.*—*1st.* In all the animals in whom it has been posssible to study the terminations of the motor nerves, there has been discovered a special organ termed motor plate (plaque motrice) at the extremity of the *axis cylinder*.

*2d.* The union of the nervous element with the muscular fasciculus is accomplished in the following manner:

When the muscular fasciculus is provided with sarcolemma, and the nervous element with its sheath, this latter is fused into the envelope of the primitive muscular fasciculus at the point where the nervous element meets the muscular fasciculus. At this point, or a little in advance of it, the medullary substance terminates, whilst the *axis cylinder* pursues its way, and penetrates into the motor plate.

*3d.* The motor plate is placed under the sarcolemma. It ordinarily presents the form of a cone whose summit is directed toward the nerve tube, whilst the base rests upon the primitive muscular fibres.

*4th.* This plate is formed of two layers superposed and very distinct, especially in those animals provided with large plates. The substance of the superior plate is granular: that of the lower layer is perfectly homogeneous, and is probably nothing more than an expansion of the *axis cylinder.*

*5th.* Within the thickness of the third granular layer of the plate, is found in the torpedo, a system of canals in which the *axis cylinder* ramifies, forming a network with large meshes. These canals are limited to a sheath which constitutes their walls.

*6th.* When the muscular fasciculi present central canals the granular substance of the plate is continuous with the granular substance contained in these canals.

*7th.* In animals provided only with slender muscular fibres, the *axis cylinder* traverses the granular substance of the plate by subdividing into two filaments which terminate in points with two extremities of the contractile element.

*8th.* Everything induces the belief that each primitive muscular fasciculus possesses but one motor plate. In this latter one or more nervous elements originate from the subdivision of the same nervous tube.

*9th.* The diameter of the motor plate augments in proportion to the size of the primitive muscular fasciculus.

